# Contact lens rehabilitation following repaired corneal perforations

**DOI:** 10.1186/1471-2415-6-11

**Published:** 2006-03-14

**Authors:** Jeewan S Titiyal, Rajesh Sinha, Namrata Sharma, V Sreenivas, Rasik B Vajpayee

**Affiliations:** 1 Rajendra Prasad Centre for Ophthalmic Sciences, All India Institute of Medical Sciences, New Delhi, India

## Abstract

**Background:**

Visual outcome following repair of post-traumatic corneal perforation may not be optimal due to presence of irregular keratometric astigmatism. We performed a study to evaluate and compare rigid gas permeable contact lens and spectacles in visual rehabilitation following perforating corneal injuries.

**Method:**

Eyes that had undergone repair for corneal perforating injuries with or without lens aspiration were fitted rigid gas permeable contact lenses. The fitting pattern and the improvement in visual acuity by contact lens over spectacle correction were noted.

**Results:**

Forty eyes of 40 patients that had undergone surgical repair of posttraumatic corneal perforations were fitted rigid gas permeable contact lenses for visual rehabilitation. Twenty-four eyes (60%) required aphakic contact lenses. The best corrected visual acuity (BCVA) of ≥ 6/18 in the snellen's acuity chart was seen in 10 (25%) eyes with spectacle correction and 37 (92.5%) eyes with the use of contact lens (p < 0.001). The best-corrected visual acuity with spectacles was 0.20 ± 0.13 while the same with contact lens was 0.58 ± 0.26. All the patients showed an improvement of ≥ 2 lines over spectacles in the snellen's acuity chart with contact lens.

**Conclusion:**

Rigid gas permeable contact lenses are better means of rehabilitation in eyes that have an irregular cornea due to scars caused by perforating corneal injuries.

## Background

Corneal scars following perforating corneal injuries cause significant visual reduction, mainly because of the direct obscuration of rays by the opacity and also because of the irregular corneal astigmatism that results due to scar. Most of these patients require penetrating keratoplasty for an optimal visual gain. Rigid gas permeable (RGP) contact lens which can mask significant amount of irregular astigmatism can correct visual morbidity in some of these patients [1-4]. In fact, in developing countries with paucity of donor material, contact lenses are considered as the first choice of optical rehabilitation in eyes with corneal scar following repaired corneal perforation.

The aim of this study was to evaluate the role of rigid gas permeable contact lens in improving the visual outcome over spectacles in scars caused by perforating corneal injuries.

## Methods

Patients presenting with corneal scars caused by perforating corneal injuries to the cornea services of the Rajendra Prasad Centre for Ophthalmic Sciences were enrolled for the study after obtaining clearance from the institute ethics committee. An informed consent was obtained from all the enrolled patients. The corneal laceration was repaired at an earlier date either at our centre or the patient was referred from some other centre after repair. Some of these patients were not improving significantly with spectacle correction and were referred for corneal transplantation. A detailed work-up was performed at our centre, which included uncorrected visual acuity (UCVA), best spectacle corrected visual acuity (BSCVA), slit lamp biomicroscopy, presence of sutures, integrity of anterior segment structures, whether the eye was phakic, aphakic or peudophakic.

Ophthalmoscopy (Direct & Indirect) was performed to rule out presence of any co-existing fundus pathology. All the sutures were removed if present and these patients were called 2 weeks after suture removal for contact lens trial. Keratometry and videokeratography was performed for the contact lens specifications. Rigid gas permeable contact lens of high DK value (Fluoroperm 92) was fitted in all the eyes by trial and error method. The lenses were fit slightly flatter than usual and a contact lens with large overall diameter of 9.5 mm to 10.5 mm was selected to achieve superior alignment fit. Contact lens fitting was performed according to refraction and keratometric values. To start with, the flattest keratometric (K) value was taken as the initial base curve of the contact lens. If the mires were grossly distorted on keratometry, corneal topographic K value at 3 mm zone was taken into consideration. The fit was evaluated on parameters of centration, movement and coverage and the fluorescein pattern under the trial lens to achieve the best fit. Over refraction was performed to achieve final power of the contact lens. The best contact lens corrected visual acuity (BCLCVA) was recorded.

In subsequent follow-up visits after prescribing contact lens, the fit was evaluated, best contact lens corrected visual acuity was recorded and presence of any contact lens related complication was ruled out. The patients were followed up for 6 months. The contact lens fit was considered successful if there was improvement in the visual acuity over spectacles, and the patient was able to wear the contact lens for at least 8 hours a day. No patient reported with any contact lens related complication during the follow up period.

The data were analyzed statistically and a comparative analysis of the visual outcome was performed on the basis of site of opacity (Central, Paracentral & Peripheral) and the lens status (Phakic, Aphakic & Pseudophakic).

### Statistical analysis

Descriptive statistics i.e. mean, standard deviation and frequency distribution was calculated for all the variables in the study. A comparison of the visual outcome obtained by contact lens and glasses in all the 40 eyes was performed by paired 't' test. To see the significant difference in the outcome between the different sites of scars and the status of lens, one way analysis of variance (ANOVA) was used. P-value of less than 0.05 was considered as statistically significant. All the calculations were performed using STATA 8.0 statistical software.

## Results

Forty eyes of 40 patients that had undergone surgical repair for post traumatic corneal perforations were fitted high DK rigid gas permeable contact lenses for visual rehabilitation. The mean age of the patients was 16.40 ± 9.04 years and 28 (70%) patients were males. The corneal scar was found to be central (1) in 18 (45%) eyes, paracentral (2) in 18 (45%) eyes and peripheral (3) in 4 (10%) eyes (Table 1). Twenty-four eyes (60%) required aphakic contact lenses (Table 1). The mean keratometric astigmatism at the time of contact lens trial was 4.58 ± 2.45D in eyes with central scar (n = 18), 2.79 ± 1.18D in eyes with paracentral scar (n = 18) and 1.87 ± 0.92D in eyes with peripheral and limbal scar. The difference in the amount of astigmatism between eyes with central scar (1) and paracentral (2) & peripheral (3) scar was found to be statistically significant (1 vs 2: p = 0.007; 1 vs 3: p = 0.012). The best corrected visual acuity of ≥ 6/18 in the snellen's acuity chart was seen in 10 (25%) eyes with spectacle correction. However, BCVA of ≥ 6/18 was possible in 37 (92.5%) eyes with the use of contact lens. This difference was found to be statistically significant (p < 0.001). The best-corrected visual acuity with spectacles was 0.20 ± 0.13 while the same with contact lens on day 1 was 0.58 ± 0.26. A comparative analysis of improvement in visual acuity with contact lens over spectacle correction was found to be statistically significant (p < 0.001). A comparative analysis of visual outcome was performed in subgroups formed on the basis of various types of corneal scars and lens status. It was found that in all these subgroups the improvement in visual acuity with contact lens over spectacle correction was found to be significant (Table 1). All but one eyes showed an improvement of BCVA by ≥ 2 lines over spectacles in the snellen's acuity chart with contact lens. One patient (2.5%) discontinued use of contact lens because of intolerance.

**Table 1:** Visual improvement with contact lens in eyes with repaired corneal laceration

**Nature of Corneal Opacity**	**BCVA with Contact Lens**	**BCVA with Spectacles**	**P value**
Central (n = 18)	0.47 ± 0.22	0.14 ± 0.09	p < 0.001
Paracentral (n = 18)	0.68 ± 0.27	0.24 ± 0.14	p < 0.001
Limbal (n = 04)	0.62 ± 0.28	0.29 ± 0.16	p < 0.001
**Lens Status**			
Aphakic (n = 24)	0.48 ± 0.21	0.14 ± 0.09	p < 0.001
Phakic (n = 07)	0.72 ± 0.34	0.28 ± 0.18	p < 0.001
Pseudophakic (n = 09)	0.74 ± 0.20	0.31 ± 0.11	p < 0.001

## Discussion

The presence of an irregular astigmatism in eyes with repaired corneal perforations prevents optimal improvement in visual acuity with spectacle correction. The scattering of light and the irregular refraction due to the presence of the scar produces increased glare sensitivity, reduced contrast sensitivity and mesopic vision apart from variable reduction in visual acuity. Rigid gas permeable (RGP) contact lenses improve visual acuity by providing a smooth refracting surface negating the irregular astigmatism due to scar [5-7]. The tear film underneath the contact lens may also neutralize surface irregularities. Study has shown that RGP contact lenses provide significant improvement in visual acuity in eyes with nebular and nebulo-macular corneal opacity [8]. Study has reported that improvement in visual function is directly related to improvement in visual acuity [9]. However, another study has shown that there is more improvement in visual acuity with RGP contact lens than in glare acuity, contrast sensitivity and mesopic vision [8]. There is no study implicating RGP contact lens in decreased contrast sensitivity though the smaller diameter of these lenses and possible edge effect may contribute to glare sensitivity [6]. Unlike RGP lenses, prolonged use of soft contact lenses has been shown to affect visual function [6,10-13].

In the present study, we found that the improvement in BCVA was much better with contact lens than spectacle correction in all types of corneal scars (central/paracentral/peripheral). The BCVA of ≥ 6/18 was seen in significantly higher number of patients with contact lens. In the present study, the corneal astigmatism at the time of contact lens fitting was more in eyes with central scar. Hence, these eyes had least UCVA and BCVA with spectacles as well as contact lens. This is understandable because the central scar comes in line with the visual axis and causes direct obstruction of rays resulting in marked scattering of rays. However, the improvement in visual acuity with RGP contact lenses over spectacles in these eyes was also significant. This could be perhaps because of the anterior contact lens surface acting as the major refracting surface. In aphakes, the visual improvement with spectacles and contact lens was not as good as phakic and pseudophakic eyes. This was perhaps because of greater severity of trauma to these eyes. However, the improvement with contact lenses in these eyes was significantly greater over spectacle correction. For aphakes, the added advantage could be the less amount of unilateral magnification induced by contact lens in comparison to spectacles. The role of contact lens to treat unilateral aphakia is well known [14-16]. These patients may tolerate contact lens better than other aphakic patients because they are usually younger.

The contact lens acceptance was very high and only one patient developed intolerance to it. We preferred a flatter fit in these patients with careful attention to air or fluorescein patterns with trial lens fittings. A flatter fit seems to be tolerated better by the cornea in long term. Moreover, there is some amount of upper lid catch of the contact lens during each blink which is acceptable in such cases. However, the factor of stability of the contact lens has to be considered while going for flatter fit.

We preferred to fit the contact lens after complete suture removal as presence of sutures can make the lens unstable as well as increase the risk of microbial keratitis.

## Conclusion

This study suggests that rigid gas permeable contact lens is an effective means of visual rehabilitation in corneal scars caused by perforating corneal injuries and should be preferred over spectacle correction.

## Competing interests

The author(s) declare that they have no competing interests.

## Authors' contributions

JST performed contact lens fitting and evaluated the patients, RS performed data collection, NS wrote the manuscript, VS performed statistical evaluation, RBV designed the study. All authors read and approved the manuscript.

## Pre-publication history

The pre-publication history for this paper can be accessed here:


